# Draft Metagenome-Assembled Genome Sequences of Three Novel Ammonia-Oxidizing *Nitrososphaera* Strains Recovered from Agricultural Soils in Western Colorado

**DOI:** 10.1128/mra.00360-22

**Published:** 2022-08-08

**Authors:** Arsen Yerlan, Rebecca A. Daly, Reza Keshavarz Afshar, Michael Shaffer, Kelly C. Wrighton, Bridget B. McGivern

**Affiliations:** a Department of Soil and Crop Science, Colorado State University, Fort Collins, Colorado, USA; b Western Colorado Research Center, Fruita, Colorado, USA; Indiana University, Bloomington

## Abstract

Microbial nitrification is critical to nitrogen loss from agricultural soils. Here, we report three thaumarchaeotal metagenome-assembled genomes (MAGs) representing a new species of *Nitrososphaera*. These genomes expand the representation of archaeal nitrifiers recovered from arid, agricultural soils.

## ANNOUNCEMENT

Ammonium- or urea-based fertilizers are the dominant form of applied nitrogen in agricultural systems (1). Ammonia-oxidizing bacteria and archaea (AOA) are recognized as partially controlling the fate of this nitrogen through nitrification, rapidly converting ammonium to nitrate, which is more mobile and can lead to substantial nitrous oxide (N_2_O) production (1–3). The AOA are classified as *Thermoproteota* (formerly *Thaumarchaeota*) and have been primarily tracked by marker gene sequencing in soil (4). Here, we report three metagenome-assembled genomes (MAGs) for a novel *Nitrososphaera* species recovered from agricultural soil in western Colorado.

Soil samples were collected from the Western Colorado Research Center (Fruita, CO, USA; 39°10′47.9994″, −108°42′0″) in February 2021. Surface (0- to 5-cm) soil samples were taken from fallow agricultural plots managed under conventional tillage (*n* = 1) and an untilled system (*n* = 1). DNA was extracted from 0.4 g of each soil using the Zymo Quick-DNA fecal/soil microbe microprep kit, following the soil protocol. Metagenomic libraries were prepared using the Tecan Ovation Ultralow v2 system and were sequenced on the NovaSeq 6000 platform on a S4 flow cell at Genomics Shared Resource, Colorado Cancer Center (Denver, CO, USA). The untilled and tilled metagenomes comprised 37.9 Gbp and 28.5 Gbp of 150-bp paired-end reads, respectively. For each metagenome, the read quality was determined using FastQC v0.11.2 (5), the reads were trimmed using Sickle v1.33 (pe -t sanger) (6) and assembled using MEGAHIT v1.2.9 (–k-min 31 –k-max 121 –k-step 10 –mem-flag 1) (7), and the contigs were binned using MetaBat2 v2.12.1 (8). The MAG quality was assessed using CheckM v1.1.2 (9), and the taxonomy was assigned using GTDB-tk v1.5.0 (r202) (10). MAG annotation was performed using DRAM (11) within KBase (12). Default parameters were used unless noted. Two of the *Nitrososphaera* MAGs (WCRC_1 and WCRC_3) were recovered from the conventional tilled soil metagenome and the other (WCRC_2) from the untilled soil metagenome.

**FIG 1:**
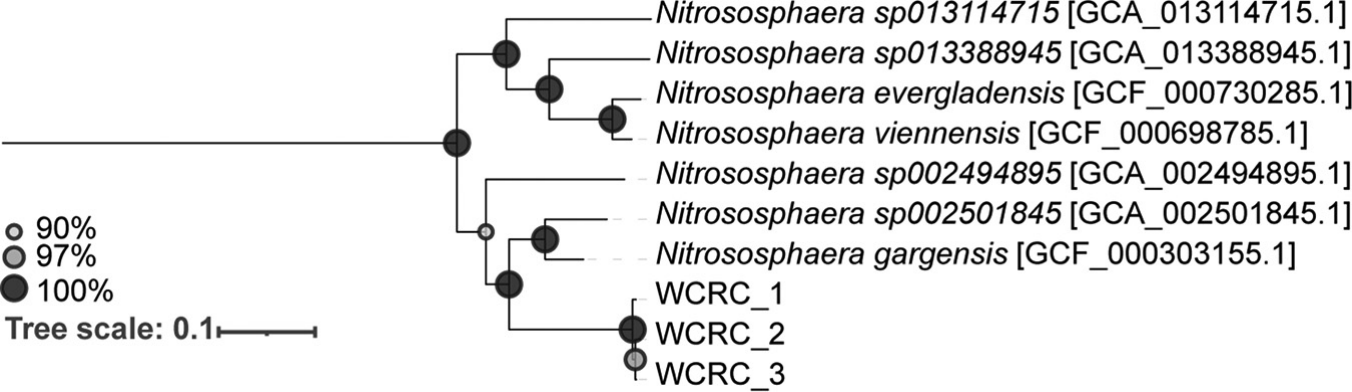
Phylogenetic tree based on the WCRC *Nitrososphaera* MAGs and GTDB-tk r202 *Nitrososphaera* species representatives. The tree is rooted on the species representatives of g_Nitrosocosmicus. The GTDB-tk de_novo_wf workflow was used to generate a multiple-sequence alignment (MSA) using g_Nitrosocosmicus as the outgroup and filtering to g_Nitrososphaera. The resulting MSA was used to construct a maximum likelihood phylogenetic tree using RAxML v8.2.9 (15) with the PROTGAMMAWAG model and 100 bootstraps. Bootstraps for the nodes were all greater than 90% and are sized according to the legend.

The three MAGs were assigned to a new species in the genus *Nitrososphaera* using GTDB-tk, where there are currently 14 *Nitrososphaera* genomic representatives across seven species (GTDB-tk r202) (Fig. 1). The pairwise amino acid identity is 97.67% between the three MAGs, suggesting that they are members of the same species (13). The statistics of these three MAGs are presented in Table 1.

**TABLE 1:** Metagenome-assembled genome statistics for WCRC_1, WCRC_2, and WCRC_3

Characteristic	Data for strain:		
	WCRC_1	WCRC_2	WCRC_3
Origin soil management	Conventional till	Untilled	Conventional till
BioSample accession no.	SAMN26177291	SAMN26177292	SAMN26177293
Genome size (bp)	1,081,057	1,177,263	876,390
No. of contigs	154	181	123
GC content (%)	44.6	44.4	44.7
Longest contig (bp)	29,717	25,749	20,557
*N*_50_ (bp)	8,096	7,090	7,633
Completeness (%)	78.8	83.5	71.36
Contamination (%)	1.94	2.91	0.97
No. of predicted coding genes	1,275	1,395	1,050
No. of tRNAs	28	25	20
Encoded rRNA	5S		
Mean base coverage (×)	6.05	6.28	10.04

Genome annotation of the *Nitrososphaera* MAGs supported their roles as AOA. All MAGs encoded the B and C subunits of ammonia monooxygenase (EC 1.14.99.39). While the A subunit is missing across the MAGs, this is likely due to the known challenge of assembling this gene and the incomplete nature of these MAGs (Table 1). In accordance with other observed *Thaumarchaeota*, hydroxylamine oxidase was absent across the MAGs, while WCRC_1 encoded nitrite reductase (14). Collectively, these MAGs provide genomic context for a novel species of ammonia-oxidizing *Nitrososphaera* derived from agricultural soils.

### Data availability.

The sequencing data for this project have been deposited at GenBank under BioProject accession number PRJNA725542. The MAGs have been deposited under BioSample accession numbers SAMN26177291, SAMN26177292, and SAMN26177293. The metagenomic reads have been deposited in the Sequence Read Archive under accession numbers SRS11831377 and SRS11831378.
